# Behaviour of Human Erythrocyte Aggregation in Presence of Autologous Lipoproteins

**DOI:** 10.1155/2012/261736

**Published:** 2011-09-08

**Authors:** C. Saldanha, J. Loureiro, C. Moreira, J. Martins e Silva

**Affiliations:** ^1^Instituto de Medicina Molecular, Unidade de Biologia Microvascular e Inflamação, Instituto de Bioquímica Faculdade de Medicina da Universidade de Lisboa. Av Prof. Egas Moniz, 1649-028 Lisboa, Portugal; ^2^Departmento de Cardiologia, Hospital Fernando da Fonseca. IC19, 2720-276 Amadora, Portugal; ^3^Departmento de Medicina I, Faculdade de Medicina da Universidade de Lisboa. Av Prof. Egas Moniz, 1649-028 Lisboa, Portugal; ^4^Instituto de Biopatologia Química Faculdade de Medicina da Universidade de Lisboa. Av Prof. Egas Moniz, 1649-028 Lisboa, Portugal

## Abstract

The aim of this work was to evaluate *in vitro* the effect of autologous plasma lipoprotein subfractions on erythrocyte tendency to aggregate. Aliquots of human blood samples were enriched or not (control) with their own HDL-C, LDL-C, or VLDL-C fractions obtained from the same batch by density gradient ultracentrifugation. Plasma osmolality and erythrocyte aggregation index (EAI) were determined. Blood aliquots enriched with LDL-C and HDL-C showed significant higher EAI than untreated aliquots, whereas enrichment with VLDL-C does not induce significant EAI changes. For the same range of lipoprotein concentrations expressed as percentage of osmolality variation, the EAI variation was positive and higher in presence of HDL-C than upon enrichment with LDL-C (*P* < 0.01). Particle size, up to LDL diameter values, seems to reinforce erythrocyte tendency to aggregate at the same plasma osmolality (particle number) range of values.

## 1. Introduction

There is scientific agreement that a high serum level of low-density lipoproteins cholesterol (LDL-C) is a risk factor for atherosclerosis and cardiovascular diseases [1–3]. A linear relationship between LDL-C levels and the occurrence of coronary artery disease is well documented in two meta-analysis [4, 5]. Conversely, it has been shown that HDL-C when at normal or high serum levels acts as a vascular protector and consequently without contribution such as a risk factor for atherosclerosis [6]. However, if its antioxidant capacity is diminished in patients with systolic heart failure, it will predict a higher risk of incident long term for adverse cardiac events [7].

Several clinical studies evidenced associations between complex lipid macromolecules; for example, high LDL-C concentrations and blood rheological behaviour, like blood hyperviscosity, that are both referred to as cardiovascular risk factors [8–10]. Blood viscosity is dependent on macro-(hematocrit and plasma viscosity) and micro-(erythrocyte deformability and aggregation) hemorheological parameters. Disturbances in blood rheological behaviour, such as high values of the blood and plasma viscosity and increased erythrocyte aggregation tendency, have been described in patients with ischemic heart diseases [11]. Red blood cells (RBCs) participate in acute coronary occlusion, mainly under conditions of lower shear rate, for example, within the microcirculation in the peri-infarct domain of myocardium [12].

Under *in vitro* stasis conditions, RBCs in normal human blood form loose aggregates with a characteristic morphology, similar to a stack of coins. Such aggregation is frequently named as rouleaux formation [13]. After prolonged stases, individual rouleaux can cluster, thereby forming three-dimensional structures, [14, 15]. Under circulation, the attractive forces involved are relatively weak, and aggregates can be dispersed during flow by the shear rate [16]. RBCs aggregation increasing at low shear rate affects blood viscosity and microvascular flow dynamics being markedly enhanced in several clinical states [17–21].

Factors influencing RBCs aggregation can be divided into (i) extrinsic factors such as levels of plasma proteins (e.g., fibrinogen, lipoproteins, macroglobulins, or immunoglobulins), hematocrit, and shear rate, and (ii) intrinsic factors, for example, RBCs shape, deformability and membrane surface properties [22–32]. RBC membrane surface properties and structure, such as surface charge and the ability of macromolecules to penetrate the membrane glycocalyx, greatly affect aggregation for cells suspended in a defined medium [33, 34]. Different studies have shown that hyperlipoproteinemia is associated with erythrocyte hyperaggregation [35–37]. The inverse correlation of erythrocyte aggregation with HDL2-C subfraction was reported in hypercholesterolemia middle-aged male population without apparent symptoms of cardiovascular disease [38]. It was evidenced *in vitro* that LDL-C enhances the RBCs aggregation induced by fibrinogen according to two aggregation models [39]. Considering the particle-like nature of the lipoproteins we raise the hypothesis that increased amounts of lipoprotein particles may change plasma osmolality with repercussions in erythrocyte aggregation.

The aim of our work was to study *in vitro* the erythrocyte aggregation tendency in blood samples collected from healthy male adults and enriched with their own plasma lipoproteins subfractions.

## 2. Material and Methods

### 2.1. Blood Samples

On consecutive days, venous blood samples were obtained with previous consent from healthy fasting volunteers adult males (*n* = 10) after 15 min in the recumbent position and collected (for two plastic tubes) with anticoagulant (10 I.U. of heparin/mL or 0.1% EDTA).

### 2.2. Lipoprotein Fractions

Lipoproteins fractions were prepared by a discontinuous NaCl/KBr density gradient ultracentrifugation using an SW 50.1 rotor (Beckman) [40]. Lipoprotein fractions were characterised by electrophoresis (Electra HR Helena Laboratories) buffer tris-barbital-sodium buffer pH 8.8) in cellulose acetate by comparison with serum controls (Lipotrol, Helena Laboratories).

### 2.3. Erythrocyte Aggregation Index

Erythrocyte aggregation was determined using the MA1 aggregometer from Myrenne GMBH (Roetgen, Germany). The MA1 aggregometer consists of a rotating cone plate chamber which disperses the sample by high shear rate of 600 s^−1^ and a photometer that determines the extent of aggregation. The intensity of light (emitted by a light emitting diode) is measured after transmission through the blood sample. The aggregation was determined in stasis for 10 seconds after dispersion of the blood sample [41].

### 2.4. Plasma Osmolality

Plasma osmolality was determined with the Osmomat 030 Cryoscopic Osmometer from Gonotec (Berlin, Germany).

### 2.5. Experimental Design

Blood samples from each donor were divided on aliquots, and after centrifugation and small volumes of plasma (0, 5 *μ*L, 10 *μ*L, 20 *μ*L, and 40 *μ*L) were discharged and replaced by equal values of their own previously enriched lipoprotein subfractions prepared a day before. With this procedure, no hematocrit variations were obtained. Blood aliquots were gently mixed by inversion, and erythrocyte aggregation was assessed. At the end of each assay, the aliquots were centrifuged at 12000 rpm for 1 minute in the Biofuge 15 centrifuge from Heraeus, and plasma osmolality was determined. HDL-C, LDL-C, and VLDL-C concentrations were expressed as percentage of osmolality variation values.

### 2.6. Statistical Analysis

The statistical evaluation performed utilized the “one-way” ANOVA with homogeneity test, cluster analysis, and average method.

## 3. Results

The major plasma lipoprotein subfractions were obtained by the discontinuous density gradient centrifugation between the density range of 1.006 and 1.300 g/mL. Each lipoprotein fraction was well banded with VLDL-C at the top, LDL-C in the upper middle and the HDL-C in the lower middle portion of the tube. After that, each fraction was pooled and submitted to electrophoresis where their obtained migration was confirmed by comparison with the serum control.

**Figure 1:**
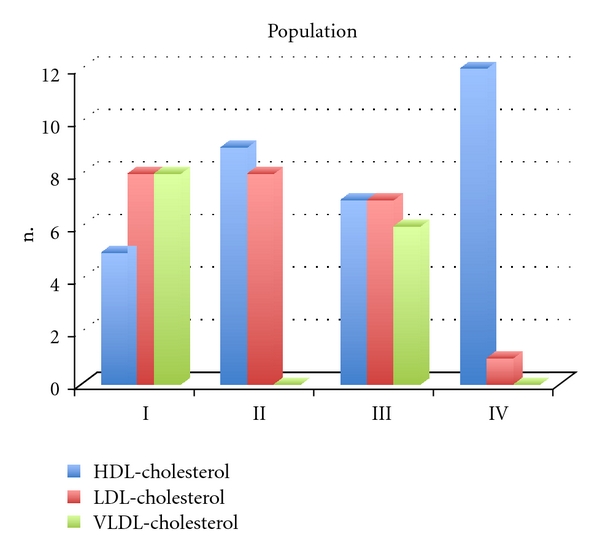
Histogram presenting the concentrations of enriched blood samples with LDL-C (red), HDL-C (blue), VLDL-C (green) distributed by four classes according the osmolality scale of values (class I 0.005–0.025; class II 0.030–0.035: class III 0.045–0.055; class IV 0.077–0.095).

The volume of each lipoprotein sub-fraction added to the autologous blood samples aliquots caused variation of osmolality concentrations in relation to its absence. Using the cluster analysis and the beverage method, four classes of concentration range expressed as osmolality variation were grouped for each LDL-C; the VLDL-C and the HDL-C enriched blood aliquots, namely, Class I 0.005–0.025; Class II 0.030–0.035; Class III 0.045–0.055; Class IV 0.077–0.095 (Figure 1).

**Figure 2:**
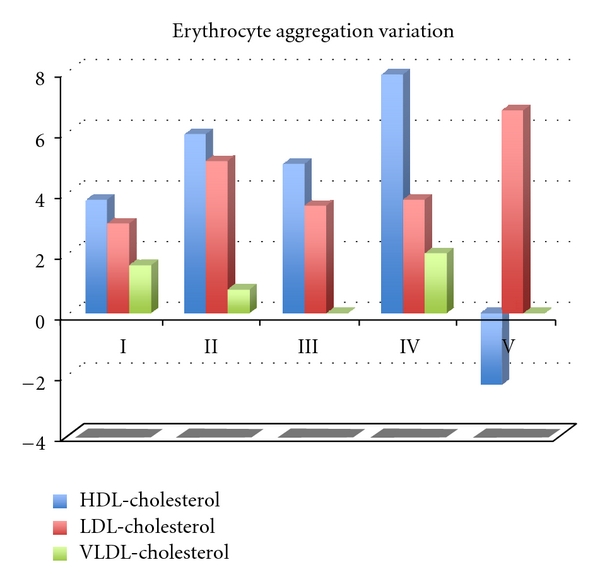
Histogram presenting the values of the erythrocyte aggregation variation obtained in enriched blood samples with LDL-C (red), HDL-C (blue), VLDL-C (green) in the four classes of concentration according the osmolality scale of values (I 0.005–0.025; II 0.030–0.035: III 0.045–0.055; IV 0.077–0.095).

**Figure 3:**
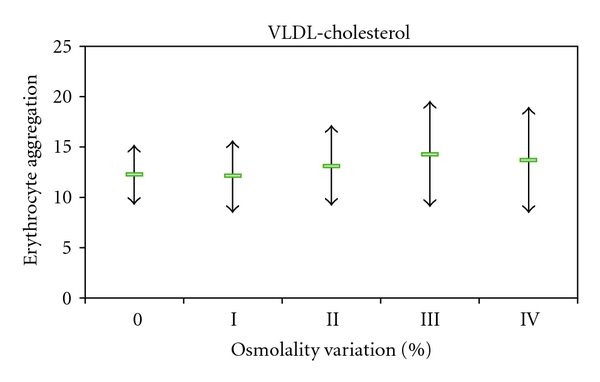
Values of erythrocyte aggregation index (mean +/− sd) obtained in enriched blood samples with HDL-C and without enrichment.

**Figure 4:**
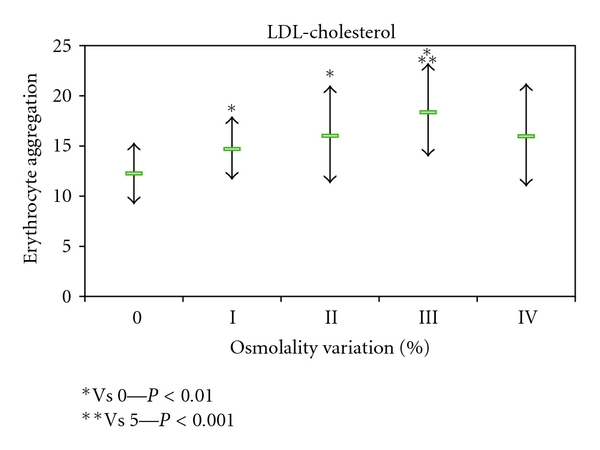
Values of erythrocyte aggregation index (mean +/− sd) obtained in enriched blood samples with HDL-C and without enrichment

**Figure 5:**
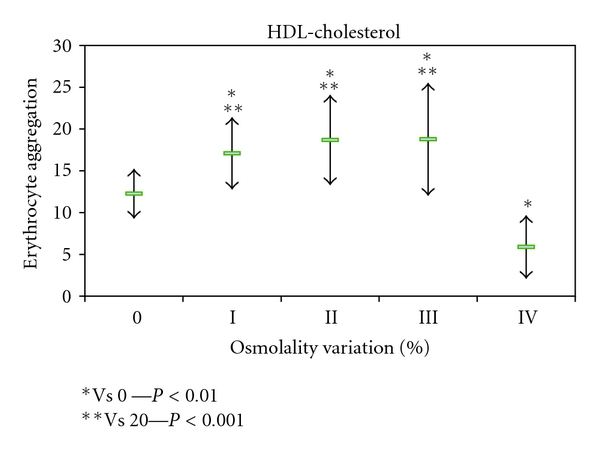
Values of erythrocyte aggregation index (mean +/− sd) obtained in enriched blood samples with LDL-C and without enrichment.

**Figure 6:**
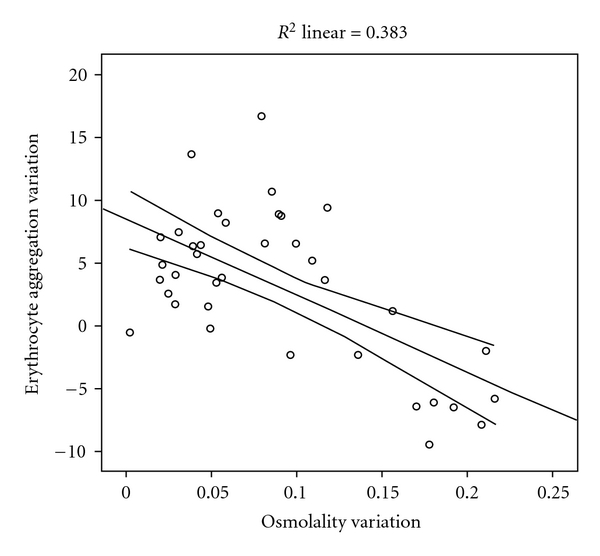
Presentation of the association obtained between the erythrocyte aggregation values and the osmolality values in enriched blood sample with HDL-C.

The erythrocyte aggregation variation for each enriched LDL, HDL, and VLDL blood aliquots in relation to the initial value is grouped by the different osmolality class variation values (Figure 2). The variation of erythrocyte aggregation in relation to the initial values depends on the plasma osmolality values, as well as of the type of lipoprotein sub-fraction. The enriched VLDL-C blood samples aliquots do not induce statistical significant variation on erythrocyte aggregation values (Figure 3). At variance in relation to the initial erythrocyte aggregation values, the enriched LDL-C blood samples presented significant statistical enhanced values at all range of percentage of osmolality variation (Figure 4). The same behaviour was verified in the enriched HDL-C blood aliquots, with exception for the higher values of percentages of osmolality variation, where a very significant (*P* < 0.0001) decrease was obtained for the erythrocyte aggregation (Figure 5). For the same range of osmolality variation, the two types of lipoproteins LDL-C and HDL-C induced different variation of erythrocyte aggregation (*P* < 0.01). When the values of osmolality variation were plotted against the respective values of aggregation variation obtained in each enriched HDL-C aliquot, a significant (*R* ^2^ = 0.383) inverse linear regression was obtained (Figure 6).

## 4. Discussion

In the present *in vitro* study, we investigated the induction of human erythrocyte tendency to aggregation by autologous lipoproteins sub-fractions. With the amount of lipoprotein sub-fractions added, changes in plasma osmolality were observed as a consequence of the increase number of particles. We used cluster method on the values of osmolality to define four classes (Figure 1).

In blood aliquots enriched with VLDL-C, no changes in erythrocyte aggregation index were verified (Figure 3). For the same amount of particles corresponding to the same class of osmolality variation, both HDL-C and LDL-C enrichment induce enhancement of erythrocyte aggregation (Figures 4 and 5). VLDL has higher diameter than the other two lipoprotein classes [42] and may either rest in the plasma bulk not interfering with erythrocyte aggregation tendency. LDL-C particles bind in a nonabsolute specific way with erythrocyte membrane, while 60% of membrane area can be occupied by HDL-C as has been described [43]. The occupancy of some areas of erythrocyte membrane by HDL-C or LDL-C, causing some interference in the promotion of EAI tendency, may be an explanation for our results. Significant (*P* < 0.01) higher values of erythrocyte aggregation were obtained in HDL-C-enriched aliquots more than for LDL-C-enriched ones at the same class of osmolality variation (under the same number of particles). The exception was for the HDL-C-enriched aliquots with the highest percentage of osmolality variation which significantly (*P* < 0.0001) decreased EAI to lower values than the aliquot control obtained. We raise the hypothesis that the higher number of unbinding HDL-C particles may increase the ionic strength to a threshold and consequently pull away the erythrocytes decreasing their tendency to aggregate.

Our results suggest that in healthy human blood aliquots enriched with autologous HDL-C or LDL-C, (where fibrinogen is present at normal range), when submitted to shear rate to disaggregate RBCs, and stopped after that, EAI tendency increases. The association between EAI values and HDL-C obtained in our experimental design is in accordance with others studies [39], which is a decrease of fibrinogen-induced RBCs aggregation in presence of the HDL2 subclass as not observed in a different experimental approach.

Recently [44], it was verified that the LDL particles number must be considered as an indicator for atherosclerosis risk factor, which has been previously remembered by others in the prevention of cardiovascular disease [45]. 

Two models, the cross-bridging and the depletion layer, were described to explain the reversible erythrocyte aggregation process at physiological conditions [46], but there are controversies and difficulties to adopt one or reject an other. The depletion model developed for polymer solutions of dextran demonstrated that there is an optimal molecular weight value to reach the greater erythrocyte aggregation tendency [47]. The hydrodynamic radius (Rh) determined for VLDL [48] is in the range between 15 nm and 40 nm which is much above of others belonging to macromolecules promoters of erythrocyte aggregation increase [49]. Particle size is an influent factor that may explain the absence of VLDL effect in the erythrocyte aggregation obtained. Regarding our results, they may also fit the cross-bridging model if, by an unknown mechanism, we assume that the particles numbers and size favour fibrinogen binding in a similar way as previously reported for immunoglobulin effects on fibrinogen-mediated erythrocyte aggregation [50, 51]. More studies are needed to explain in what model fit the effects of lipoproteins in erythrocyte aggregation. Our results may contribute to better understand the direct associations between high erythrocyte aggregation tendency and other cardiovascular risk factors such as hyperlipoproteinemia.
